# The salvage therapy utilizing human umbilical cord-derived mesenchymal stem cells for the treatment of critically ill patients with COVID-19

**DOI:** 10.3389/fimmu.2025.1594373

**Published:** 2025-07-04

**Authors:** Weiqi Yao, Boran Li, Yingan Jiang, Qiaoyu Yuan, Wenjie Wu, Ruizhen Hou, Qi Qi, Haibo Dong, Yun Zhang, Yu Zhang

**Affiliations:** ^1^ Wuhan Optics Valley Vcanbio Cell & Gene Technology Co., Ltd., Wuhan, Hubei, China; ^2^ Wuhan Optics Valley Vcanbiopharma Co. Ltd., Wuhan, Hubei, China; ^3^ Hubei Engineering Research Center for Human Stem Cell Preparation, Application and Resource Preservation, Wuhan, China; ^4^ School of Materials Science and Engineering, Wuhan University of Technology, Wuhan, Hubei, China; ^5^ Department of Infectious Diseases, Renmin Hospital of Wuhan University, Wuhan, China; ^6^ VCANBIO Biological Cell Storage Service (Tianjin) Corp., Ltd, Tianjin, China; ^7^ Institute of Hematology and Blood Diseases Hospital, Chinese Academy of Medical Sciences and Peking Union Medical College, Tianjin, China; ^8^ State Industrial Base for Stem Cell Engineering Products, Tianjin, China; ^9^ VCANBIO Cell & Gene Engineering Corp., ltd., Tianjin, China

**Keywords:** COVID-19, human umbilical cord-derived mesenchymal stem cells, inflammation, lung injury, therapeutic options

## Abstract

**Background:**

The therapeutic options for patients with coronavirus disease (COVID-19) are limited. Mesenchymal stem cells (MSCs) have immunomodulatory and regenerative properties that may inhibit excessive inflammatory responses, promote recovery from COVID-19-induced lung injury, and potentially serve as a therapeutic option.

**Methods:**

An evaluation of the safety and efficacy of using human umbilical cord mesenchymal stem cells (hUC-MSCs) in 5 critically ill patients with COVID-19 was conducted in this study. In all patients, hUC-MSCs were administered intravenously three times at a dosage of 3 × 10^7^ cells per time, 2 days between each infusion. Safety was evaluated using adverse events. The efficacy was assessed by coagulation function (serum D dimer), inflammatory index (CRP and IL-6) and immune index (lymphocyte count, neutrophil count, CD4, CD19, and CD16 + 56), as well as chest computed tomography (CT) images. Post-infusion visits focused on oxygen saturation and progression of lung lesions.

**Results:**

Infusions of hUC-MSCs were not associated with any serious adverse events. The CT scans indicate that lung lesions have been adequately controlled after receiving the hUC-MSCs. HUC-MSCs injection improved immune system function and alleviated inflammation.

**Conclusions:**

According to our findings, intravenous infusion of hUC-MSCs has proven to be safe, and it has also proven to show potential therapeutic benefits for patients with severe COVID-19.

## Introduction

In Wuhan, the city at the heart of the outbreak of the novel coronavirus (COVID-19), SARS-COV-2, in December 2019 (1–3). At the time of this writing, COVID-19 is still spreading and has yet to be effectively controlled in many countries. As of July 2024, there were over 775 million confirmed cases of COVID-19 around the world (4). The World Health Organization (WHO) declared COVID-19 as a pandemic and classified the outbreak as a public health emergency of international concern (PHEIC) (5).

SARS-CoV-2 mainly infects the respiratory tract and lungs, causing severe organ damage and pneumonia, which include severe acute respiratory syndrome (ARDS) and even death. Excessive inflammatory responses and cytokine storms are considered to be the major causes of organ damage, driving the progression of the disease among severe or critically ill patients (6–9). Besides, the therapy of direct antiviral therapy and supplemental oxygen (5), as immune-modulation-related therapeutic methods, has become a potential choice for preventing the progression of COVID-19 and improving the condition of COVID-19 patients (10). So far, several immunotherapy methods have been used for the treatment of COVID-19, including convalescent plasma therapy, anti-interleukin (IL-6) receptor antibody, glucocorticoid and adoptive cell therapies (5, 11–13). However, their side effects and efficacy are a matter of clinical concern, so the search for more effective treatments with fewer side effects is imperative.

Mesenchymal stem cells (MSCs) have the characteristics of self-renewal, multi-directional differentiation potential, immune regulation, and tissue repair (11, 12), which have been widely used in clinical trials to treat diseases of autoimmune, endocrine, and infectious diseases (13–17). MSCs have also been demonstrated to have good safety and efficacy in the treatment of acute lung injury (ALI), idiopathic pulmonary fibrosis disease (IPF), influenza virus infection, and other lung diseases (18–21). Therefore, this study aims to investigate the hypothesis that MSCs could inhibit the inflammatory response and lung damage caused by COVID-19 infection. In this study, we report the results of salvage therapy using human umbilical cord-derived MSCs (hUC-MSCs) for five severe COVID-19 patients, which was carried out at the very start of the COVID-19 outbreak in Wuhan.

## Materials and methods

### Participants

**Figure 1:**
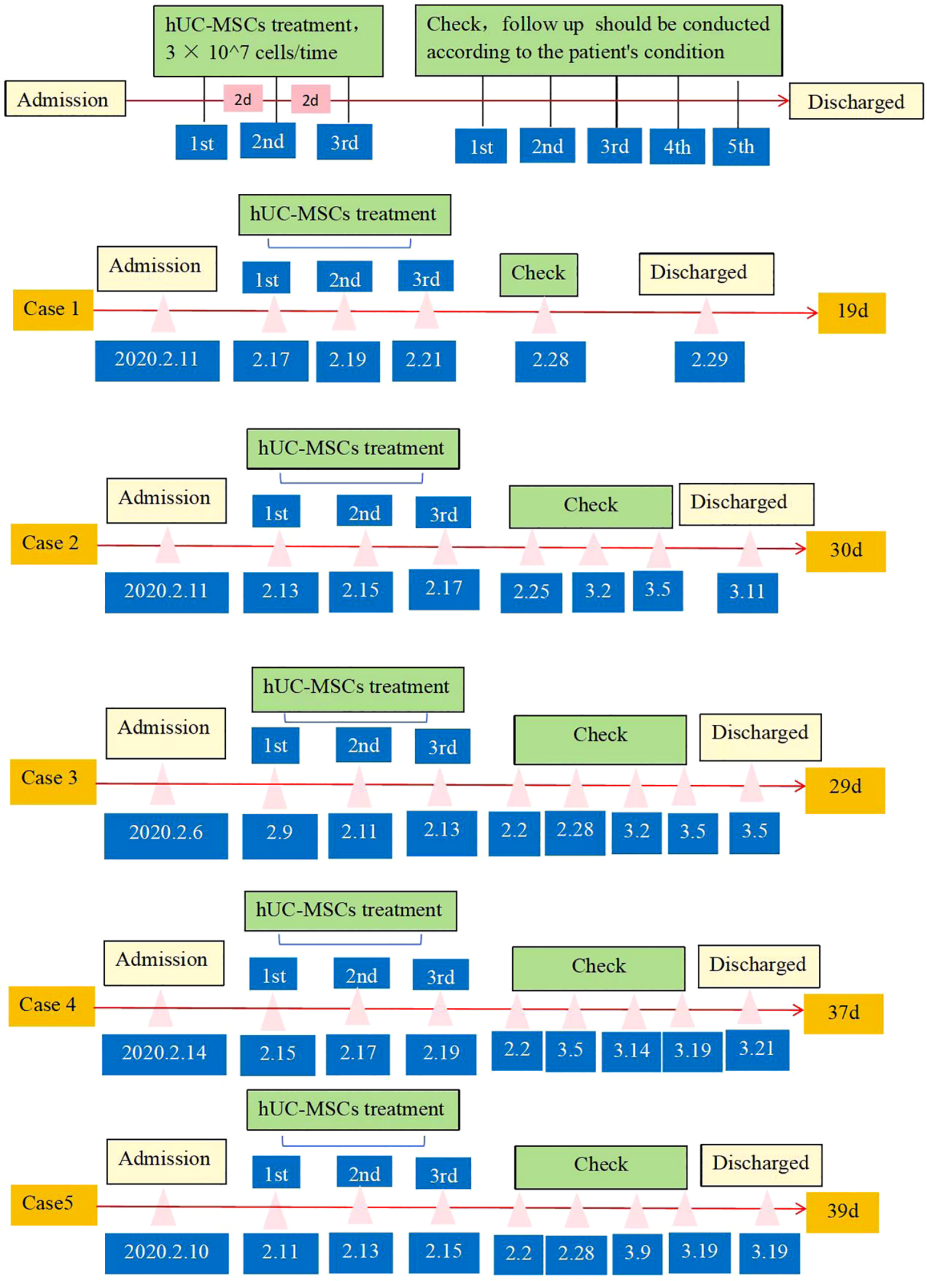
The flow chart of the MSC transplantation treatment of patients. Five patients were infused with MSCs 3 times (3 × 10^7^ cells/time), underwent five examinations.

This study was implemented between February and March 2020 in Renmin Hospital of Wuhan University, China. All five patients who took part in the study were diagnosed with the severe COVID-19 type based on the Diagnosis and Treatment Protocol for Severe and critical cases of COVID-19 (Version 2) (China National Health Commission). This study was supported by the ethics committee of Renmin Hospital of Wuhan University (No: WDRY2020-K016). Before the experiment, all five patients signed informed consent including provisions for the use of data for scientific research. As shown in the flow chart (**Figure 1**), five patients were infused with hUC-MSCs three times (3 × 10^7^ cells/time), which referred to the hUC-MSCs Phase I clinical trial in Fifth Medical Center belonging to the PLA General Hospital during the same period (22).

### Inclusion criteria and exclusion criteria

Patients were be included if they met the following criteria: (1) Patients in hospital with severe COVID-19 with laboratory confirmed SARS-CoV-2 infection by reverse transcription polymerase chain reaction (RT-PCR) aged 18–75 years old; (2) chest computed tomography (CT) imaging definitely pneumonia combined with lung damage. In addition, patients with any of the following conditions were reckoned as severe cases: (1) dyspnea (respiratory rate ≥ 30 times/min); (2) oxygen saturation (SpO_2_) of 93% or lower indoor air. The exclusion criteria consisted of patients with shock or COVID-19 combined with any one of other organ failures, patients with any malignant tumor, those who received invasive ventilation, pregnancy or breastfeeding, or co-infection of other pathogens.

### Cell preparation and transplantation

hUC-MSCs from male bodies who were delivered at full-term (with the consent of parents of UC donors), as previously reported, were gathered by VCANBIO Cell & Gene Engineering Corp, Ltd (23). Briefly, hUCs were soaked in 75% ethanol for 10 s, then the blood vessels were rinsed with phosphate-buffered saline (PBS) three times. Isolation and culture of hUC-MSCs were performed under a laminar flow hood in sterile conditions. The cord was cut into 1 to 2 cm pieces and dissected carefully to expose underlying UC tissues. Wharton’s Jelly (WJ) tissues were then cut into 2-mm^3^ pieces from the cord tissue and planted upside down in culture flasks (75 cm^2^) in 4 mL growth medium consisting of DMEM/F12, supplemented with fetal bovine serum (10% FBS, BI, Israel) at 37°C with 5% CO_2_ for 4 h. Then, 10 mL supplementary medium was added to the flasks. The complete medium was changed every 4 to 5 days until cells reached 60 to 80% confluence. Tissue explants were removed after 8–10 days of culture. Detached the adherent cells with 1×TrypLE (GIBCO, USA) and then re-plated at a density of approximately 6-8×10^3^ cells per cm^2^ for further expansion. Master and working cell banks were set superlatively, and a homogenous population of these cultured cells at passage 5 (P5) was used for all the experiments in this study. Harvested cells were detected according to the minimal criteria suggested by the International Society for Cellular Therapy (ISCT) (11) and showed fibroblast-like morphology with the ability of plate attachment. Cell surface markers, including CD19, CD34, CD11b, CD45, CD73, CD105, CD90 and HLA-DR, were analyzed by flow cytometry (BD, FACS Calibur, USA). The control IgGs were from isotype-matched normal mice, and we acquired and saved all Images and data. Cell viability was detected using trypan blue and 7-AAD/Annexin V by flow cytometry after preparation and before administration. The cell product passed the certification of the National Institutes for Food and Drug Control of China.

### Laboratory tests, imaging and clinical outcomes

The laboratory tests, including blood tests, to determine the coagulation function, oxygenation index, inflammatory index, immune index, and nucleic acid of COVID-19 were tested at ** Hospital of ** University. Patients underwent chest computerized tomography (CT) examination after admission and treatment. Other clinical information about the patients was obtained from their medical records. The clinical results were evaluated comprehensively according to the following aspects: (1) respiratory symptoms, such as cough and wheezing; (2) non-oxygen saturation, oxygenation index, blood routine, inflammation indicators and other laboratory tests; and (3) lung imaging findings.

### Statistical analysis

Some frequency tables and graphs were used to describe the individual data. Because the size of the study population was insufficient to enable a conclusive statistical analysis, the sample size was not calculated based on statistical power calculations.

## Results

### Baseline characteristics of the enrolled patients

The 5 subjects enrolled in this study all underwent nucleic acid testing for SARS-CoV-2 during hospitalization, with positive test results confirming their diagnosis as severe COVID-19 patients. All subjects received hUC-MSCs therapy on D1, D4, and D7 after enrollment. Baseline characteristics of all patients, including age, gender, vital signs on admission, temperature, PaO_2,_ SpO_2_, co-existing co-morbidities, duration of hospitalization and anti-SARS-CoV-2 IgM/IgG antibodies at hospitalization were recorded (**Table 1**). One week after the final dose (D7), all 5 subjects were retested for SARS-CoV-2 nucleic acid. Only 1 subject tested positive, while the remaining 4 subjects turned negative. The testing results of COVID-19 nucleic acid are shown in **Table 2** (The relevant data in the table were partial test results of patients after hUC-MSCs treatment, and did not include positive test results at admission limited by the condition at that time).

**Table 1:** Baseline characteristics of enrolled patients with COVID-19.

Patient ID	Case 1	Case 2	Case 3	Case 4	Case 5
Age (years)	65	69	62	64	75
Gender	Female	Female	Male	Male	Female
Vital signs on admission					
RR (breaths/min)	21	17	21	17	19
PR (beats/min)	71	51	78	69	67
SBP (mmHg)	124	112	138	120	95
DBP (mmHg)	61	62	75	102	50
Temp(°C)/	36.5	36.2	38.5	36.3	39
SpO_2_	98%	93%	91%	91%	91%
Comorbidities	None	Type 2 diabetes mellitus and coronary heart disease	None	None	None
Duration of hospitalization (days)	19	29	35	38	46
Antibody at hospitalization					
IgM antibody (AU/ml)	75.73	77.31	139.86	66.73	58.72
IgG antibody (AU/ml)	8.42	3.57	16.59	7.26	28.41

**Table 2:** Detection of novel coronavirus nucleic acid.

Detection of novel coronavirus nucleic acid	SARS-CoV-2 testing status	D1 (First dose of hUC-MSCs)	Date	Nasopharyngeal swab	Anal swab
Case 1	Positive 2.11	2.17	2.21	Negative	Negative
			2.25	Negative	Negative
Case 2	Positive 2.11	2.13	2.21	Negative	NA
			2.22	NA	Positive
			2.24	Positive	NA
			2.25	NA	Positive
			2.28	Negative	Positive
			3.01	Negative	NA
			3.09	Negative	NA
Case 3	Positive 2.6	2.9	2.19	Negative	NA
			2.21	Negative	NA
			2.22	Negative	Negative
			2.28	Negative	Negative
Case 4	Positive 2.14	2.15	2.16	Positive	NA
			2.19	Negative	NA
			2.22	Negative	Negative
			2.25	Negative	Negative
			2.28	Negative	Negative
Case 5	Positive 2.10	2.11	2.16	Negative	NA
			2.21	Positive	NA
			2.25	Negative	NA
			2.28	Negative	Negative
			3.02	Negative	Negative

### CT scans and clinical outcomes

**Figure 2:**
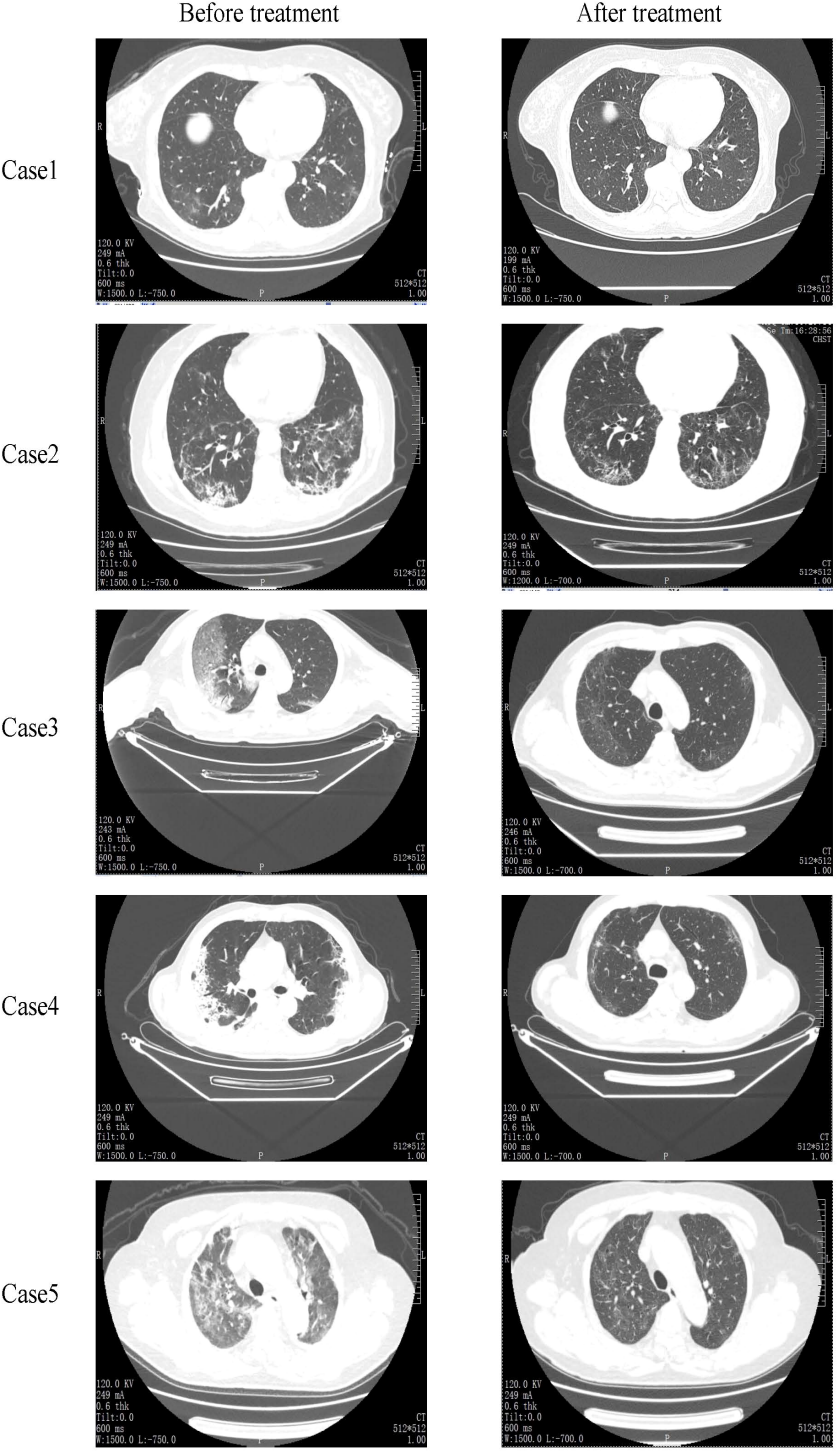
Chest CT images of the case patients pre and post treatment. After hUC-MSCs transfusion, CT scans revealed the absorption of pulmonary pathological changes and well control of lung lesions.

**Figure 3:**
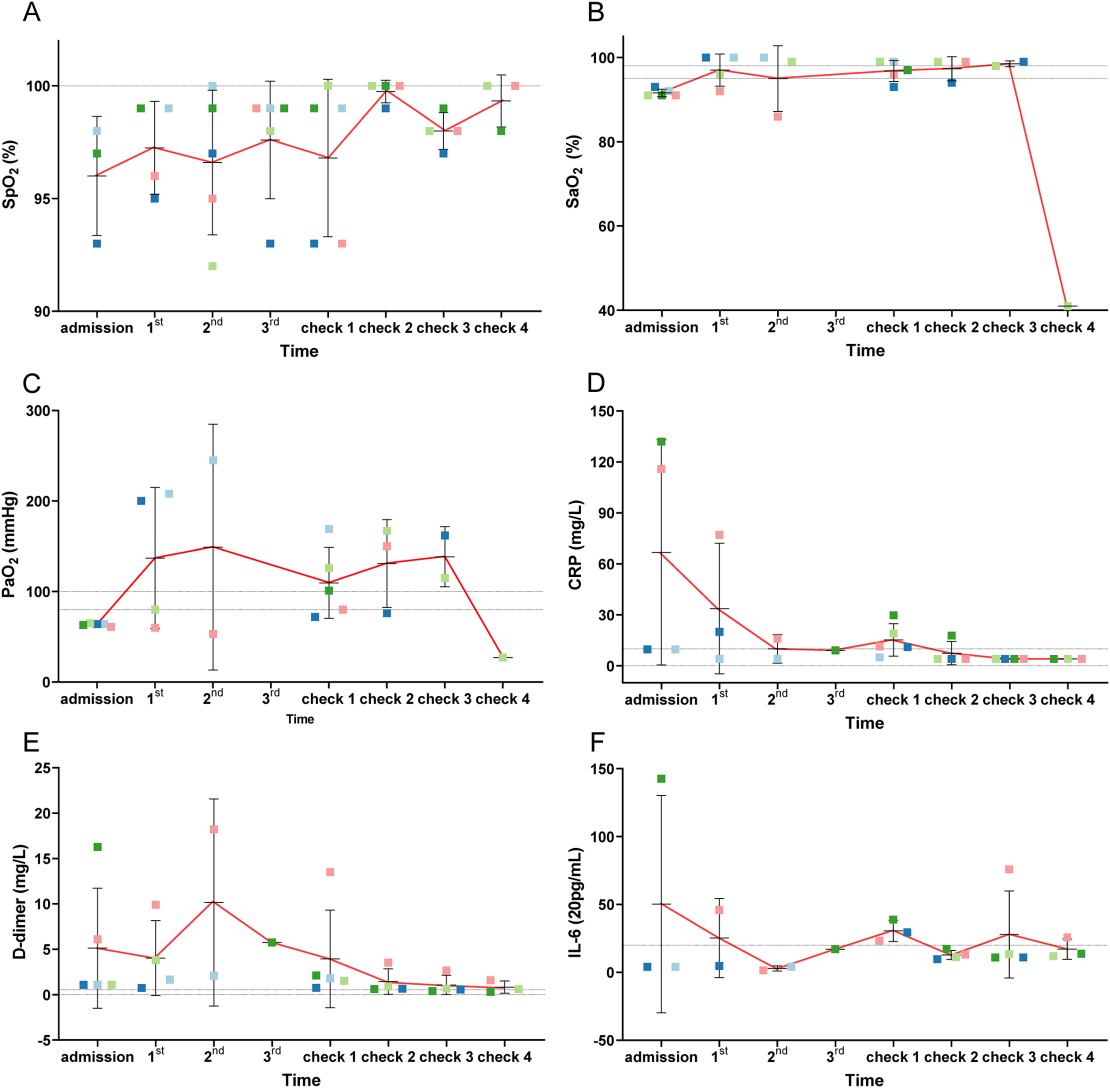
Changes of several indexes (SpO_2_, SaO_2_, PaO_2_, CRP, D-dimer, IL-6) of patients pre and post treatment. They were SaO_2_ **(A)**, SpO_2_ **(B)**, PaO_2_ **(C)**, CRP **(D)**, D-dimer **(E)** and IL-6 **(F)** respectively. From the analysis of blood gas results, due to severe pneumonia caused by SARS-CoV-2 infection, the subjects' SpO_2_, SaO_2_, and PaO_2_ were at low levels prior to treatment. Following hUC-MSCs therapy, with the control of the inflammatory response and absorption of lung lesions, lung function gradually recovered, accompanied by gradual improvements in SpO_2_, SaO_2_, and PaO_2_. Concurrently, it was observed that after hUC-MSCs administration, CRP, D-dimer, and IL-6 levels progressively decreased alongside the immunomodulatory effects and suppression of the inflammatory response.

**Figure 2** lists the CT scan results of all five patients. All patients showed ground glass opacity bilateral lung infection, and viral pneumonia before treatment. After hUC-MSCs transfusion, CT scans revealed the absorption of pulmonary pathological changes and well control of lung lesions. Case 3 indicated bilateral pleural thickened, adherent, slightly larger mediastinal lymph nodes before cell therapy. After treatment, no prominent enlarged lymph nodes were found in the mediastinum, and the lesion size and density of pulmonary lesions decreased. Case 4 and case 5 had infections disappeared whole lung infections vanished and the density was lower than pre-treatment. In our study, the alleviation of respiratory symptoms including cough and wheezing was found after the treatment of hUC-MSCs, but the detailed changes in symptom intensity were not recorded due to the urgency of treatment of severe patients. For all severe patients, the blood oxygen saturation and partial pressure of arterial oxygen were improved after hUC-MSCs treatment (**Figures 3A–C**).

### Laboratory examination

**Figure 4:**
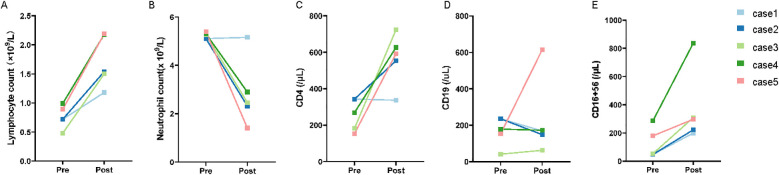
Immune indicators in patients with COVID-19. They were Lymphocyte count **(A)**, Neutrophil count **(B)**, CD4 **(C)**, CD19 **(D)**, and CD16+56 **(E)** respectively. From the trend of changes in the lymphocyte lineage (CD4, CD19, CD16+56), the levels were relatively low before administration, consistent with characteristics of the SARS-CoV-2 infected population. Following hUC-MSCs therapy, as the condition improved and inflammatory responses were controlled, the lymphocyte lineage demonstrated a recovery trend. Regarding neutrophil trends, elevated IL-6 and IL-8 caused by virus-mediated inflammatory responses stimulated neutrophil recruitment and proliferation, resulting in higher pre-administration neutrophil levels. MSCs exhibit immunomodulatory effects, and after hUC-MSCs therapy reduced immune responses to regulate neutrophil reduction.

Laboratory tests included coagulation function (serum D dimer), inflammatory index (CRP and IL-6), and immune index (lymphocyte count, neutrophil count, CD4, CD19, and CD16 + 56) (**Supplementary Tables S1–S5**). In the study, the count of lymphocyte counts, CD4, CD19, and CD16 + 56 were increased (**Figures 4A, C–E**), and neutrophil count was decreased in most patients during the treatment (**Figure 4B**), indicating the improvement in immune system function after cell therapy with hUC-MSCs. When the dynamics of a large panel of inflammatory cytokines (including C-reactive protein (CRP) and interleukin-6 (IL-6) were simultaneously monitored in hUC-MSCs-treated patients, we found there was a reduced trend in the levels of all these cytokines within the therapy (**Figures 3D, F**). Of course, other inflammatory factors such as IL-2, IL-4, IL-10, cytokines TNF, IFN-ki and respiratory rate were also counted, but no significant differences and trends were found after hUC-MSC therapy (**Figure 1**). In this research, we also examined D-dimer in the days before and after cell therapy, as shown in **Figure 3E**. The results showed that the D-dimer levels in the subjects were elevated before administration (considered to be caused by SARS-CoV-2 infection). After completing 3 doses of hUC-MSCs, all subjects showed a decrease in D-dimer levels, which continued to decrease to levels below those observed prior to medication administration. Additionally, besides low-grade fever and nausea were reported as adverse events, hUC-MSCs treatment was not associated with any serious adverse events.

## Discussion

Similar to the infections of SARS-COV and MERS-COV (24, 25), the mortality of mild or moderate COVID-19 patients is relatively low. However, the mortality of severely and critically ill patients is high, which is the main concern when developing clinical treatment (26). Studies have shown that the immune system of COVID-19 patients, which is caused by SARS-COV-2 infection, can recover with the help of immune stimulants or on its own if the breathing difficulty of patients can be resolved as soon as possible, to successfully resist further viral invasion (27). Autopsies have revealed that excessive sputum production in COVID-19 patients may lead to severe ARDS, which is consistent with the pathological findings showing high levels of inflammatory cytokines in the patient’s lung tissues (9). Therefore, in addition to antiviral therapy, suppressing the inflammatory response in the lungs and improving respiratory function of COVID-19 patients may be the key to ameliorate symptoms and save lives (28, 29). Earlier studies have reported that MSC transplantation may promote lung repair and regulate the inflammatory process to reduce fibrosis (30, 31). Therefore, MSCs offer great potential to reduce the inflammatory response of COVID-19 patients through modulation of the immune system.

C-reactive protein (CRP) is a typical serum protein that rapidly increases in inflammatory reaction during the acute phase (27). Previous studies have shown that COVID-19 patients are more likely to manifest as decreased lymphocytes and increased CRPs (3, 28, 32), which is consistent with the results of this study. In this study, we found that patients showed increased lymphocyte count and decreased levels of inflammatory markers (CRP and IL-6), accompanied by alleviated dyspnea after hUC-MSC therapy. In view of the fact that Case2 was associated with diabetes and coronary heart disease, previous studies had found that both diabetes and coronary heart disease are accompanied by systemic or local inflammatory responses to varying degrees (33–37). Existing research results suggest that hUC-MSC could treat these two indications to a certain extent by alleviating inflammatory responses in patients with diabetes and coronary heart disease (38, 39). Therefore, although our study did not detect clinical indicators of diabetes and coronary heart disease in case2, it can be inferred from historical studies that hUC-MSC can also have therapeutic effects on diabetes and coronary heart disease to a certain extent on the basis of alleviating the pathological development of COVID-19. In addition, hUC-MSC treatment improved the blood oxygen saturation (**Figures 3A, B**) and partial pressure of blood oxygen (**Figure 3C**) to the normal ranges. Otherwise, we considered that the sao2 value detected by check4 of case3 was an outlier and was not relevant to hUC-MSC therapy, nor had it been observed in other similar studies. The patients’ CRP, D-dimer and IL-6 levels (**Figures 3D–F**) showed the same trend. Therefore, changes in these indicators may be the first response to hUC-MSC treatment and can be used as the monitoring indicators for future MSC clinical studies.

Among the five patients included in this study, the longest time from admission to discharge was 39 days, the shortest was 18 days, and the average time between admission and treatment was only 2.8 days. Based on the national epidemic situation and the environmental conditions of the hospital at that time, when the symptoms of the patient were alleviated after a period of treatment, or when the nucleic acid was negative, that was, it was considered to be discharged when the patient was not a serious case. During treatment, hUC-MSCs may have an effect on alleviating lung structural damage and inflammatory cell infiltration. Therefore, administration of hUC-MSC treatment after COVID-19 infection may be able to inhibit inflammation, thereby alleviating the symptoms and damage caused to COVID-19 patients.

In a previous work, the investigators (40) screened 101 patients to evaluate the safety and clinical benefits of MSCs in the treatment of severely ill patients with COVID-19 and found that lung lesions were significantly reduced the treatment was well tolerated. Similarly, in the present study, we did not observe any significant adverse reactions, besides low fever and nausea, which resolved spontaneously within 4 hours. Nevertheless, future comprehensive studies with long-term follow-up are needed to determine whether MSC infusion causes any lasting adverse effects. Also, due to the short follow-up of this study, we were not able to determine the tolerance and safety of MSC transplantation in these patients. But in a review of one-year follow-up reports from other similar study (41), it was found that MSCs administration improved total lung volume compared with placebo, as evidenced by a reduction in the proportion of solid lesion volume at each follow-up point, and the incidence of symptoms and CT image findings in the MSCs group were superior to those in the placebo group. After two years, the proportion of patients with 6-MWD below the lower limit of the normal range was slightly lower in the MSC group than in the placebo group. At 18 months, the SF-36 overall health score was higher in the MSC group than in the placebo group (42). Importantly, there was no difference in adverse events and tumor markers between the two groups at either 1-year or 2-year follow-up. These results were able to explain the safety and efficacy of MSCs. In addition, relevant studies had found that the effect of MSCs is particularly obvious in critically ill patients (43). 2–4 days after MSCs transplantation, symptoms such as fever and shortness of breath in critically ill patients disappear, and blood oxygen saturation returns to normal (44). Therefore, considering the results of previous studies related to the use of MSCs in the treatment of COVID-19 patients, we believe that MSCs provide a viable therapy for COVID-19 patients, especially for those who are severely ill.

This study was conducted in Wuhan from January to March 2020. At that time, Wuhan, was the center of the epidemic in China. The virus had strong infectivity and virulence in the early stage of the outbreak, and the medical system lacked sufficient understanding of COVID-19, resulting in a large number of critically ill patients after infection. The rapid treatment of MSC for critically ill patients greatly alleviates the tension of medical resources and provides important time for the formulation and improvement of follow-up standard diagnosis and treatment norms. Of course, this work had several limitations due to the suddenness and severeness of the epidemic and the urgency of diagnosis and treatment. Firstly, this study only enrolled five patients who were treated with hUC-MSC and lacked a control group. Since this was not a true clinical trial, we could neither summarize our phase 1 data from the sample nor draw conclusions about the efficacy or long-term safety of hUC-MSCs as a treatment for severe COVID-19 patients. Secondly, this study was carried out in the early stages of the COVID-19 outbreak. Due to the urgency of the situation and few acknowledge about COVID-19 at that time, there was little research and limited data in the field. Even so, this study also provided relatively important research value, and we suggest that the vital signs of body temperature, blood oxygen saturation and respiration rate should be monitored, and meanwhile the indicators related to underlying diseases should be also monitored focally for critically ill patients. In a word, MSCs may be used as one of the important candidates for COVID-19 or other severe lung diseases.

In summary, this study provides evidence for the effective treatment of COVID-19 with hUC-MSCs, which can ameliorate pulmonary function by suppressing inflammation in damaged lungs. Despite many clinical trials on the treatment of COVID-19 with UC-MSCs, the research on the effectiveness and long-term safety of UC-MSCs remains in its infancy. We consider that MSCs will be a valuable tool for the future treatment of COVID-19.

## Data Availability

The original contributions presented in the study are included in the article/**Supplementary Material**. Further inquiries can be directed to the corresponding author.
