# Supplementary material for: The salvage therapy utilizing human umbilical cord-derived mesenchymal stem cells for the treatment of critically ill patients with COVID-19
Source: Front Immunol. 2025 Jul 4;16:1594373. doi: 10.3389/fimmu.2025.1594373 (PMC12271227; doi:10.3389/fimmu.2025.1594373)
Supplement: Supplementary file 1 [file DataSheet1.docx]

**Table S1.** Case 1 laboratory inspection data

| **Case 1** | **2.11** | **2.17**  **(msc)** | **2.19**  **(msc)** | **2.22**  **(2.21 msc)** | **2.28** |
| --- | --- | --- | --- | --- | --- |
| WBC (3.5-9.5)10^9 /L | 6.34 | 8.36 | 9.86↑/7.42 | 5.05 | 6.77 |
| NEUT% (40-75)%;  NEUT#(1.86.3)*10^9/L | 80.5↑(5.11) | 85.6↑(7.15↑) | 92.3↑(9.1↑) | 3.02 | 5.16 |
| LYM% (20-50)%;  LYM#(1.1-3.2)*10^9/L | 11.4↓(0.72↓) | 9.8↓(0.82↓) | 6.4↓(0.63↓) | 1.59 | 1.18 |
| D-dimer (0-0.55) mg/L | 1.07↑ | 1.65↑ | 2.09↑ | 2.14↑ | 1.8↑ |
| FDP (0-5) mg/L | 3.85 | 6.7↑ | 7.3↑ | 7.04↑ | 4.33 |
| ALT(9-50)U/L | 96↑ | 19.00 | 25 | 53↑ | 17.00 |
| AST(15-40)U/L | 37 | 16.00 | 26 | 45↑ | 15.00 |
| TBIL(0-23) μmol/L | 8.30 | 7.50 | 5.7 | 8.3 | 9.90 |
| BUN(3.1-8.0)μmol/L | 7.58 | 3.50 | 4.16 | 4.61 | 2.66↓ |
| CREA(57-97)μmol/L | 73.00 | 40↓ | 41 | 54 | 48.00 |
| CK(50-310)U/L | 26↓ | 22↓ | 20↓ | 17↓ | 21↓ |
| CRP(0-10)mg/L | 9.7 | <5.0 | <5.0 | 5 | 5.00 |
| PaCO_2_(35-45)mmHg | 34↓ | 43 | 44 | 42 | 48↑ |
| PaO_2_(80-100)mmHg | 64↓ | 208↑ | 245↑ | 193↑ | 169↑ |
| Oxygenation (95-98)% | 93↓ | 100↑ | 100 | 100 | 99.00 |
| CD4%(33-58)%;  CD4#404-1612/μL | 43.64(343↓) | 29.43↓(222↓) | 88↓ | 576 | 337↓ |
| CD19%(5-22)%;  CD19# 80-616/μL | 236 | 118 | 102 | 342 | 169 |
| CD16+56%(5-26)%; CD16+56#84-724/μL | 5.81(48↓) | 21.53(152) | 253 | 162 | 199 |
| IL-6 <10pg/mL | 4.07 |  | 4.18 | 6.7 |  |
| IL-10≤5.9pg/ml |  |  | 4.44 | 3.79 |  |

**Table S2**. Case 2 laboratory inspection data

| **Case 2** | **2.11** | **2.13**  **(msc)** | **2.16**  **(2.15 msc)** | **2.18**  **(2.17 msc)** | **2.25** | **3.2** | **3.5** |
| --- | --- | --- | --- | --- | --- | --- | --- |
| WBC (3.5-9.5)10^9 /L | 6.34 | 10.16↑ | 6.18 | 8.17 | 4.57 | 5.15 | 4.63 |
| NEUT% (40-75)%;  NEUT#(1.86.3)*10^9/L | 80.5↑  (5.11) | 86.6↑  (8.8↑) | 68.1  (4.21) | 81.6↑  (6.67↑) | 2.54 | 3.28 | 2.32 |
| LYM% (20-50)%;  LYM#(1.1-3.2)*10^9/L | 11.4↓  (0.72↓) | 7.8↓  (0.79↓) | 19.4↓  (1.2) | 11.4↓  (0.93↓) | 1.17 | 1.21 | 1.54 |
| D-dimer (0-0.55) mg/L | 1.07↑ | 0.75↑ | 0.51 | 0.69↑ | 0.77↑ | 0.67↑ | 0.58↑ |
| FDP (0-5) mg/L | 3.85 | 4.97↑ | 4.83↑ | 2.82 | 2.45 | 1.13 | 1.93 |
| ALT(9-50)U/L | 96↑ | 56.00 | 36 | 34 | 21 | 20 | 20 |
| AST(15-40)U/L | 37 | 18.00 | 19 | 17 | 20 | 20 | 21 |
| TBIL(0-23) μmol/L | 8.30 | 8.80 | 7.6 | 9.9 | 9.4 | 7.7 | 9.5 |
| BUN(3.1-8.0)μmol/L | 7.58 | 5.30 | 5.6 | 6 | 3.4 | 3.23 | 3.77 |
| CREA(57-97)μmol/L | 73.00 | 66.00 | 70 | 60 | 72 | 73 | 70 |
| CK(50-310)U/L | 26↓ | 18↓ | 11↓ | 11↓ | 25↓ | 30↓ | 30↓ |
| CRP(0-10)mg/L | 9.7 | 20↑ | 7.7↑ | <5.0 | 11↑ | <5.0 | <5.0 |
| PaCO_2_(35-45)mmHg | 34↓ | 37↑ | 47↑ | 39 | 45 | 43 | 42 |
| PaO_2_(80-100)mmHg | 64↓ | 200↑ | 80 | 42↓ | 72↓ | 76↓ | 162↑ |
| Oxygenation (95-98)% | 93 | 100 | 95 | 76↓ | 93 | 94↓ | 99↑ |
| CD4%(33-58)%;  CD4#404-1612/μL | 43.64  (343↓) | 36.36  (291↓) | 45.3  (645) | 32.33↓  (273↓) | 457 | 484 | 554 |
| CD19%(5-22)%;  CD19# 80-616/μL | 236 | 122 | 277 | 165 | 160 | 168 | 149 |
| CD16+56%(5-26)%; CD16+56#84-724/μL | 5.81  (48↓) | 18.25  (130) | 8.51  (108) | 18.82  (149) | 251 | 252 | 222 |
| IL-6 <10pg/mL | 4.07 | 4.7 |  | 2.82 | 29.47↑ | 9.7 | 11.05 |
| IL-10≤5.9pg/ml |  |  |  |  | 6.7↑ | 5.91↑ | 5.32 |

**Table S3.** Case 3 laboratory inspection data

| **Case 3** | **2.6** | **2.9**  **(msc)** | **2.11**  **(msc)** | **2.13**  **(msc)** | **2.22** | **2.28** | **3.2** | **3.5** |
| --- | --- | --- | --- | --- | --- | --- | --- | --- |
| WBC (3.5-9.5)10^9 /L | 5.98 | 5.98 |  |  | 2,97↓ | 3.71 | 3.18↓ | 4.6 |
| NEUT% (40-75)%;  NEUT#(1.86.3)*10^9/L | 88.1↑  (5.27) | 88.1↑  (5.27) |  |  | 1.8 | 2.03 | 1.74↓ | 2.46 |
| LYM% (20-50)%;  LYM#(1.1-3.2)*10^9/L | 8↓  (0.48↓) | 8↓  (0.48↓) |  |  | 0.62↓ | 1.15 | 0.91↓ | 1.5 |
| D-dimer (0-0.55) mg/L | 1.08↑ | 3.8↑ |  |  | 1.53↑ | 0.91↑ | 0.68↑ | 0.61↑ |
| FDP (0-5) mg/L | 3.2 | 16.08↑ |  |  | 6.76↑ | 5.08↑ | 4.52 | 3.85 |
| ALT(9-50)U/L | 76↑ | 47 |  |  | 47 | 37 | 24 | 19 |
| AST(15-40)U/L | 62↑ | 35 |  |  | 30 | 18 | 13↓ | 16 |
| TBIL(0-23) μmol/L | 8.9 | 12.6 |  |  | 9.2 | 12.3 | 7.9 | 9 |
| BUN(3.1-8.0)μmol/L | 8.9 | 8.6 |  |  | 4.1 | 4.26 | 2.89↓ | 3.79 |
| CREA(57-97)μmol/L | 69 | 59 |  |  | 54↓ | 52↓ | 54↓ | 58 |
| CK(50-310)U/L | 53 | 23↓ |  |  | 21↓ | 31↓ | 30↓ | 32↓ |
| CRP(0-10)mg/L |  |  |  |  | 19↑ | <5.0 | <5.0 | <5.0 |
| PaCO_2_(35-45)mmHg | 40 | 36 |  |  | 44 | 43 | 40 | 58↑ |
| PaO_2_(80-100)mmHg | 65↓ | 80 |  |  | 126↑ | 167↑ | 115↑ | 27↓ |
| Oxygenation (95-98)% | 91 | 96 |  |  | 99 | 99 | 98 | 41↓ |
| CD4%(33-58)%;  CD4#404-1612/μL | 47.13  (182↓) |  | 47.62  (183↓) |  | 340↓ | 471 | 414 | 725 |
| CD19%(5-22)%;  CD19# 80-616/μL | 42↓ |  | 62↓ |  | 45↓ | 48↓ | 45↓ | 63↓ |
| CD16+56%(5-26)%; CD16+56#84-724/μL | 15.36  (53↓) |  | 10.82  (40↓) |  | 94 | 241 | 152 | 308 |
| IL-6 <10pg/mL |  |  |  |  |  | 11.19 | 13.37 | 11.94 |
| IL-10≤5.9pg/ml |  |  |  |  |  | 5.71 | 7.66↑ | 7.66↑ |

**Table S4.** Case 4 laboratory inspection data

| **Case 4** | **2.14** | **2.15**  **(msc)** | **2.17**  **(msc)** | **2.19**  **(msc)** | **2.25** | **3.5** | **3.14** | **3.19** |
| --- | --- | --- | --- | --- | --- | --- | --- | --- |
| WBC (3.5-9.5)10^9 /L | 6.76 | 6.44 | 6.03 | 4.93 | 4.94 | 4.66 | 5.02 | 5.61 |
| NEUT% (40-75)%;  NEUT#(1.86.3)*10^9/L | 78.6↑  (5.31) | 74.2  (4.78) | 72.3  (4.36) | 55.5  (2.74) | 2.71 | 53.5  (2.49) | 2.8 | 2.9 |
| LYM% (20-50)%;  LYM#(1.1-3.2)*10^9/L | 14.6↓  (0.99↓) | 17.7↓  (1.14) | 18.4↓  (1.11) | 32.9  (1.62) | 1.67 | 34.8  (1.62) | 1.73 | 2.18 |
| D-dimer (0-0.55) mg/L | 16.28↑ | 10.95↑ | 8.59↑ | 5.75↑ | 2.11↑ | 0.63↑ | 0.4 | 0.31 |
| FDP (0-5) mg/L | 46.53↑ | 29.61↑ | 4.97↑ | 14.08 | 5.92↑ | 1.58 | 1.13 | 1.23 |
| ALT(9-50)U/L |  | 79↑ | 74↑ | 60↑ | 51↑ | 26 | 16 | 14 |
| AST(15-40)U/L |  | 52↑ | 38.00 | 21 | 25 | 18 | 16 | 15 |
| TBIL(0-23) μmol/L |  | 8.5 | 6.90 | 9.3 | 8.1 | 8.4 | 9.9 | 12.3 |
| BUN(3.1-8.0)μmol/L |  | 5.22 | 5.30 | 4.81 | 3.9 | 3.04↓ | 3.72 | 3.98 |
| CREA(57-97)μmol/L |  | 69 | 61.00 | 66 | 69 | 66 | 72 | 65 |
| CK(50-310)U/L |  | 55 | 40↓ | 40↓ | 42↓ | 50 | 62 | 75 |
| CRP(0-10)mg/L | 132↑ | 16.6↑ | 17.2↑ | 9.1 | 29.8↑ | 17.8↑ | <5.0 | <5.0 |
| PaCO_2_(35-45)mmHg | 37 | 35 | 36 |  | 48↑ |  |  |  |
| PaO_2_(80-100)mmHg | 63↓ | 121↑ | 94 |  | 101↑ |  |  |  |
| Oxygenation (95-98)% | 91 | 99 | 97 |  | 97 |  |  |  |
| CD4%(33-58)%;  CD4#404-1612/μL | 33.2  (269↓) | 40.68  (461) | 41.92  (500) | 39.56  (662) | 510 | 490 | 551 | 627 |
| CD19%(5-22)%;  CD19# 80-616/μL | 179 | 225 | 270 | 21.52  (392) | 221 | 143 | 176 | 173 |
| CD16+56%(5-26)%; CD16+56#84-724/μL | 32.65↑  (286) | 18.97  (214) | 14.96  (197) | 17.5  (318) | 295 | 450 | 458 | 836↑ |
| IL-6 <10pg/mL | 142.62↑ |  | 13.39↑ | 17.01 | 38.86↑ | 17.29 | 10.91 | 13.71 |
| IL-10≤5.9pg/ml |  |  |  | 5.08 | 4.67 | 6.55↑ | 5.57 | 5.02 |

**Table S5.** Case 5 laboratory inspection data

| **Case 5** | **2.10** | **2.11(MSC)** | **2.13(MSC)** | **2.15(MSC)** | **2.21** | **2.28** | **3.9** | **3.19** |
| --- | --- | --- | --- | --- | --- | --- | --- | --- |
| WBC (3.5-9.5)10^9 /L | 6.57 | 4.03 | 4.33 | 5.32 | 5.81 | 4.7 | 5.38 | 4.14 |
| NEUT% (40-75)%;  NEUT#(1.86.3)*10^9/L | 82.1↑  (5.39) | 66.8  (2.69) | 64.5  (2.79) | 42.1  (2.24) | 3.43 | 1.8 | 33.8  (1.82)↓ | 34↓  (1.41↓) |
| LYM% (20-50)%;  LYM#(1.1-3.2)*10^9/L | 13.5↓  (0.89↓) | 24.1  (0.97↓) | 25.4  (1.1) | 47.6  (2.53) | 1.72 | 2.34 | 55.9  (3.01)↑ | 52.9↑  (2.19) |
| D-dimer (0-0.55) mg/L | 6.1↑ | 9.91↑ | 18.21↑ | 15.3↑ | 13.51↑ | 3.53↑ | 2.65↑ | 1.58↑ |
| FDP (0-5) mg/L | 6.2↑ | 5.62↑ | 3.53 | 44.9↑ | 34.08↑ | 10.44↑ | 7.3↑ | 5.64↑ |
| ALT(9-50)U/L | 36.00 | 44↑ | 26 | 27 | 24 | 26 | 20 | 14 |
| AST(15-40)U/L | 52↑ | 59↑ | 25 | 27 | 21 | 25 | 14↓ | 17 |
| TBIL(0-23) μmol/L | 7.60 | 7.2 | 6.4 | 7.8 | 9 | 4.1 | 4.4 | 7.4 |
| BUN(3.1-8.0)μmol/L | 10.5↑ | 9.4↑ | 8.9↑ | 8.3 | 6.91 | 6.33 | 7.11 | 6.28 |
| CREA(57-97)μmol/L | 134↑ | 102↑ | 87↑ | 87↑ | 82↑ | 85↑ | 85 | 76 |


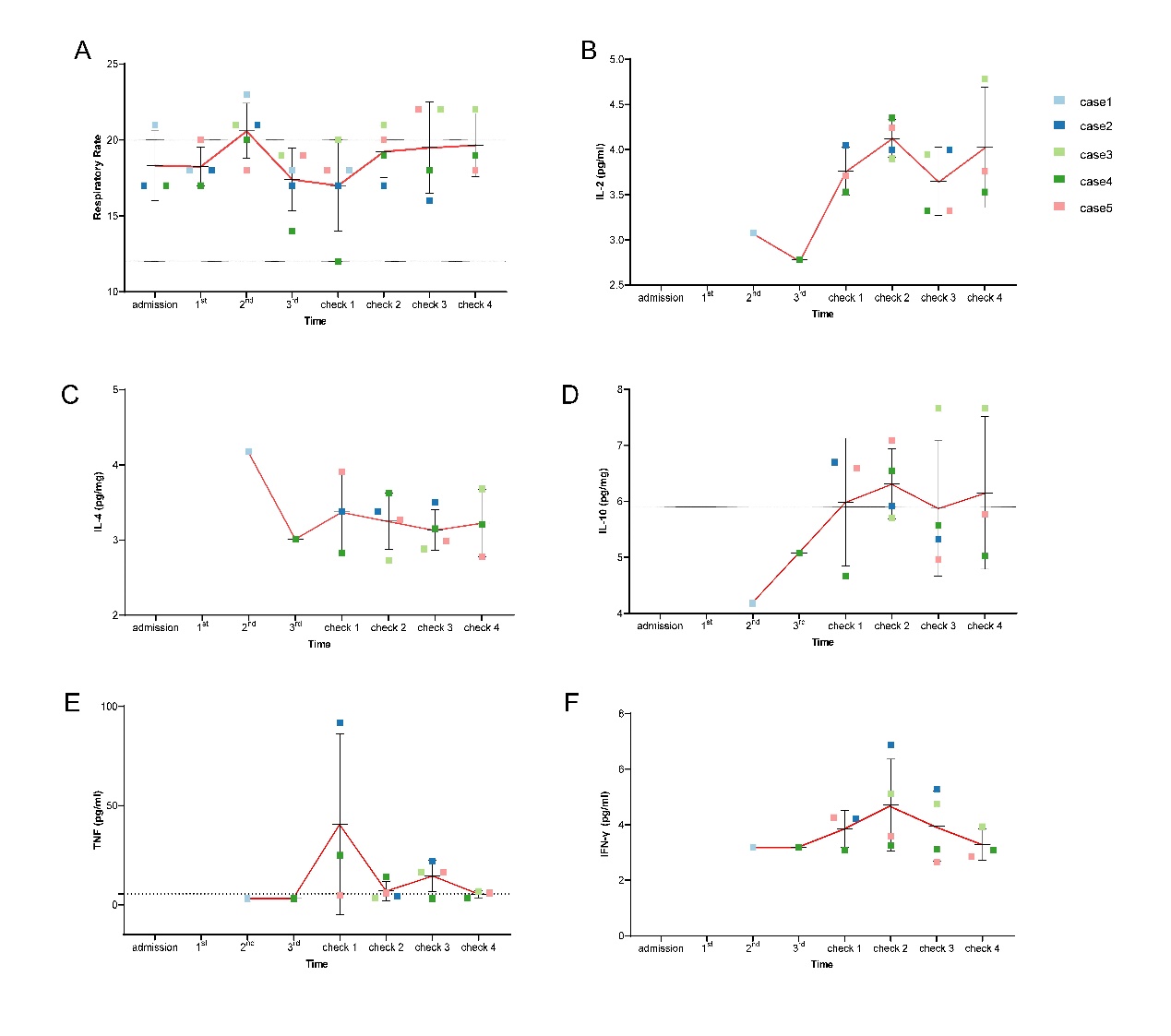


**Fig.s1** Changes of other indexes of patients pre and post treatment.
